# Impact of Disease Modifying Therapy on MS-Related Fatigue: A Narrative Review

**DOI:** 10.3390/brainsci14010004

**Published:** 2023-12-20

**Authors:** Mahmoud Elkhooly, Fen Bao, Evanthia Bernitsas

**Affiliations:** 1 Department of Neurology, Southern Illinois University School of Medicine, Springfield, IL 62702, USA; elkhoolymahmoud27@outlook.com; 2 Department of Neurology, Wayne State University School of Medicine, Detroit, MI 48201, USA; 3 Department of Neurology and Psychiatry, Minia University, Minia 61519, Egypt

**Keywords:** multiple sclerosis, fatigue, disease modifying therapies

## Abstract

Multiple sclerosis (MS) is a chronic autoimmune disease that affects the central nervous system by causing inflammation, demyelination and neurodegeneration. Fatigue is the most prevalent and one of the most disabling symptoms among people with MS (pwMS). Due to its complexity and subjective character, fatigue is still little understood despite its frequent occurrence and severe impact. The potential causes, effects, and treatments of fatigue associated with MS have been extensively studied in recent years. Though the benefits of such a variety of contributions are obvious, there have not been many attempts to evaluate the effect of disease modifying therapies (DMTs) on MS-related fatigue. In this review, we summarize clinical trials and research studies, and we discuss the effect of different DMTs on MS-related fatigue.

## 1. Introduction

One of the most prevalent and disabling signs of multiple sclerosis (MS) is fatigue, which has been linked to a lower quality of life [1,2,3]. Little is understood about the fundamental reason, despite its relevance. Numerous pharmacological and non-pharmacological modalities have been researched, but there are limited data to support their efficacy [4,5]. It was proposed that peripheral or central inflammation could be the root of fatigue [6].

Fatigue severity is self-reported. Over the years, several imaging biomarkers have been identified in an attempt to associate the subjective nature of fatigue with objective measures [7,8,9].

Fatigue remains a significant issue for most MS patients and along with depression is closely correlated with the quality of life in the MS patients [10]. Given that the pathophysiology of MS-related fatigue may involve neuroinflammation, it is reasonable to hypothesize that the DMTs with their well-known anti-inflammatory action may potentially ameliorate fatigue. However, it is unknown how these drugs affect fatigue because the duration of fatigue was not examined in randomized, prospective trials [7,8,9].

Reducing fatigue is a significant benefit that could lead to an improvement in quality of life because it may hinder daily tasks even in the early stages of MS when physical disability is not a major problem. Despite the fact that there is no cure from multiple sclerosis, many DMTs have been released over the last three decades. In this narrative review, we discuss results reported from randomized clinical trials, observational studies and meta-analysis, and we summarize the effect of DMTs, either positive or negative, on MS fatigue.

## 2. Methods

In this narrative review, PubMed, Google Scholar, and clinicaltrials.gov databases were searched using the following keywords: “multiple sclerosis”, “fatigue”, “disease-modifying therapies” and their synonyms in conjunction with Cochrane search filters for identifying trials. The searches were conducted from 1993 to October 2023.

We identified 46 articles for 13 different DMTs. Eight studies addressed natalizumab; eight focused on glatiramer acetate; twelve investigated sphingosine-1-phosphate inhibitors; seven discussed dimethyl fumarate; four examined teriflunomide; three examined interferon; and one each examined ocrelizumab, alemtuzumab, mitoxantrone, and cladribine. There were no studies exploring the effect of ofatumumab on fatigue, whereas there was one ongoing study on the impact of ozanimod on MS-related fatigue.

## 3. Fatigue and Rating Scales

Different scales have been used to measure fatigue severity. The modified fatigue impact scale (MFIS) is a modified version of the 40-item Fatigue Impact Scale (FIS), which was developed initially to evaluate how fatigue affects people with chronic diseases, notably MS, in terms of quality of life. A questionnaire containing 10 “physical” questions, 10 “cognitive” items, and 20 “social” items is used by FIS patients to score how much tiredness has interfered with their lives over the previous four weeks, with 0 denoting “no problem” and 4 denoting “severely affected”. Maximum score is 160 [1,11]. Nine “physical” items, ten “cognitive” items, and two “psycho-social” elements make up the MFIS. Higher ratings indicate a stronger influence on quality of life, with a maximum score of 84. Another scale is FSS, which was created by Krupp in 1989; it is a self-reported scale that assesses the level of fatigue. FSS contains nine items. The patient is asked to select a number between 1 and 7, where 1 denotes strong disagreement and 7 denotes strong agreement to represent how strongly the patient agrees or disagrees with each statement in each question. Severe fatigue is typically indicated by a score of 4 or higher [3].

The Fatigue Symptoms and Impacts Questionnaire–Relapsing Multiple Sclerosis (FSIQ-RMS-S) is another scale for MS fatigue severity measurement. Seven questions make up the FSIQ-RMS-S, which measures symptoms of fatigue during the preceding 24 h. Physical weakness, mental exhaustion, physical fatigue, energy, feeling worn out, feeling sleepy, and feeling worn out while at rest are the things listed. On an 11-point numerical rating scale (NRS), from 0 (no symptoms) to 10, respondents are asked to score each item (highest level of symptoms). Individual item scores are added together to produce an FSIQ-RMS-S score that ranges from 0 to 70 and is then rescaled to range from 0 to 100. More serious fatigue is indicated by a higher FSIQ-RMS-S score [12].

## 4. MS Therapies

The most typical technique to monitor MS disease progression is through routine clinical evaluations and MRI scans. It is crucial to keep track of these metrics in order to assess whether and how well a patient’s DMT is controlling their MS. For MS patients, a range of DMTs are currently available. The various administration methods, action and effect of different DMTs on fatigue are shown in Table 1 and Table 2.

## 5. Oral DMTs

### 5.1. Dimethyl Fumarate (Tecfidera)

Dimethyl fumarate (DMF) is an FDA-approved drug for relapsing forms of MS (RRMS, CIS and SPMS). DMF activates the erythroid-derived nuclear factor, a defense system against oxidative and inflammatory stress [59].

Several previous studies explore the effect of DMF on fatigue. A large prospective 12-month observational study (RESPOND) on 318 RRMS patients who switched from glatiramer acetate (GA) to DMF showed marked improvement in MFIS on DMF, from 10.7 (5.2) at baseline to 9.3 (5.2) after 12 months (mean change 0.83 [3.51]; *p* = 0.0002) [13].

Another prospective study on 34 RRMS patients showed that at 3, 6, 12, and 24 months following DMF treatment, MFIS scores were considerably reduced (7.85 ± 12.20, *p* < 0.01; 7.75 ± 15.59, *p* < 0.01; 8.40 ± 15.55, *p* < 0.001; 10.25 ± 10.92, *p* = 0.0001) [16]. Amato et al. confirmed the previous results in reduction in MFIS score over 24 months among 165 RRM patients (least square mean (LSM) difference: −3.526, 95% CI: −4.782 to −2.271; *p* < 0.001) [15]. Additionally, the PROTEC study showed that 867 of 1105 RRMS patients treated with DMTs showed marked improvement in MFIS [14].

MFIS-5 remained stable over a 12-month period of DMF treatment in the ESTEEM trial involving 2025 RRMS patients [18].

Another retrospective small sample study performed by Pandey et al. failed to show any improvement in self-reported fatigue in DMT-treated patients especially when it co-exists with depression [19].

Comi and colleagues investigated the effect of DMF on sleep quality on 223 RRMS over 48 weeks (177 RRMS on DMF, 46 RRMS on interferon). The study explored fatigue as a secondary end point using the FSS. Despite the subjective self-reported improvement in fatigue due to significant positive effect on sleep quality, the study failed to demonstrate significant changes in fatigue, mobility or quality of life over the study duration [17].

### 5.2. Sphinosine-1—Phosphate Modulators

#### 5.2.1. Fingolimod

Fingolimod is an FDA-approved DMT for CIS and RRMS also marketed as Gilenya. It has distinct immunoregulatory characteristics and affects sphingosine-1-phosphate receptors. Fingolimod stops immune cells from leaving lymphoid tissue and reaching the inflammatory tissue [60].

There are limited studies which evaluate the impact of fingolimod on fatigue. Masinque’s earlier prospective open label trial on 54 RRMS patients found no improvement in the MFIS-measured fatigue score and FSS at a 6-month interval [28].

In contrast, another study compared the effect of fingolimod with that of the injectable DMT among 1053 RRMS patients, which showed that marked improvement in FSS in the fingolimod group who received it at least 6 months after switch from injectable DMT [21,22,61]. This can be better explained by a lower mean of EDSS (2.5 versus 2.7) in Masingue’s study. The Hamburg Quality of Life Questionnaire in MS (HAQUAMS) was administered to 281 patients who were randomly assigned to take oral fingolimod (*n* = 188) or a placebo (*n* = 93). With fingolimod, the overall HAQUAMS score improved, while with placebo, it worsened. The mean score changed by 0.02 with fingolimod 1.25 mg (*p* < 0.05 against placebo), 0.01 with fingolimod 5.0 mg, and 0.12 with placebo from baseline to month six. Additionally, fingolimod 1.25 mg was superior to placebo in the HAQUAMS sub-domain for fatigue/thinking (*p* < 0.05 vs. placebo) [20]. As a side effect, all groups in FREEDOMS and TRANSFORMS experienced documented adverse events at a similar rate. Fatigue and headache were among the side effects that occurred in the fingolimod groups and affected approximately 10% of participants [26,27]. In another trial, 24 (19.7%) of the 122 patients receiving fingolimod had fatigue [25]. This was confirmed by another study conducted by Rojas [24] who reported that 16 patients of 145 (11%) of fingolimod-treated patients reported fatigue. The same percentage was also reported by Brown et al. [23]. More studies are needed for further exploration to identify patients whose fatigue will benefit from fingolimod versus those who will develop fatigue as a side effect.

#### 5.2.2. Siponimod

Siponimod is also known as Mayzent, an FDA-approved medication for CIS, RRMS and SPMS. Its lipophilic nature makes it capable of crossing the blood–brain barrier and act on sphingosine-1-phosphate. There are limited data on the relation between it and fatigue.

In prior research, the safety of Siponimod has been explored, namely in the EXPAND trial which included 1099 secondary progressive MS patients for more than 5 years. It showed that fatigue may be reported as a side effect of Siponimod use with an incidence rate of 6.3% compared to 6.6% in the placebo group [29]. Hoffman et al. reported that among 1500 patients treated by Siponimod, fatigue and quality of life remained stable after 12 months using MFSC and EQ-5D, respectively [30].

#### 5.2.3. Ponesimod

Ponesimod is a highly selective modulator of sphingosine-1-phosphate receptor 1 (S1P1) with low potential for drug–drug interaction because it is an orally active drug [62]. It also has no active metabolites. Ponesimod prevents lymphocytes from leaving lymphoid organs, which causes a quick, dose-dependent, and reversible decrease in peripheral blood lymphocyte levels [63,64].

Ponesimod was compared with teriflunomide in phase three OPTIMUM study, which included 1133 patients with RRMS. Ponesimod was significantly superior to teriflunomide in reducing fatigue using FSIQ-RRM with a mean score of −3.57 (−0.01 vs. 3.56; *p* < 0.001) in the ponesimod group [31].

#### 5.2.4. Ozanimod

Ozanimod received FDA approval in March 2020 to treat RRMS, active SPMS, and CIS. Ozanimod is a sphingosine-1-phosphate receptor (S1PR) modulator and is marketed under the trade name ZEPOSIA. The only S1PR modulator currently approved by the US FDA that does not need genetic testing or first-dose monitoring is Ozanimod, in contrast to earlier medications in its class [65,66]. The first study examined ozanimod on fatigue is the OCEAN study, which is still ongoing in Germany. It aims to include 1300 RRMMS patients over 36 months. Their primary outcomes include patient satisfaction and effectiveness. As a secondary outcome, it also aims to study fatigue and quality of life using FSMC and MSQOL-54, respectively [67].

### 5.3. Teriflunomide (Aubagio)

The FDA has approved teriflunomide for CIS and RRMS patients. It works by inhibiting mitochondrial dihydro-orotate dehydrogenase (DHODH), which results in less pyrimidine being synthesized, which is essential for the proliferation of lymphocytes. Therefore, the absence of DHODH caused by teriflunomide limits the ability of lymphocytes to attack the neurological system. Previous studies explore its effect on fatigue. A total of 324 individuals were randomly assigned to receive IFN-1a, teriflunomide 7 mg, or teriflunomide 14 mg. FIS scores showed more prominent fatigue in the INF-1a group, though differences were only significant when compared with teriflunomide 7 mg [33]. Additionally, in Teri-Real observational study, which includes 212 patients, the overall FIS score as well as its cognitive and psychosocial components remained stable. After two years, compared to the baseline, the physical component of FIS did exhibit a statistically significant improvement (11.33 vs. 13.09) [32].

Despite having a favorable outcome, Teri-FAST, which was a 2-year, prospective, observational study over 210 patients, confirmed that fatigue remained stable with no significant improvement [35]. Data from TEMSO and TOWER showed that there is an increased fatigue rate especially with relapses leading to hospitalization [34].

### 5.4. Cladribine

A deoxyadenosine analogue called cladribine is phosphorylated to form human lymphocyte-specific cytotoxin 2-chlorodeoxyadenosine triphosphate [68,69]. A prior two-year study examined how cladribine affected quality of life using the Multiple Sclerosis Quality of Life-54 (MSQOL-54) and the EuroQol five-dimension three-level (EQ-D5-3L)18. Low-dose cladribine 3.5 mg administered to 5148 RRMS patients showed marked improvement using EQ-5D but failed to show any statistical improvement using MSQOL-54 [36]. Further research is needed to reach a definite conclusion.

## 6. Injectable DMTs

### 6.1. Glatiramer Acetate (GA)

A polypeptide-based medicine called glatiramer acetate (GA) (Copolymer-1, Copaxone, Teva, Israel, YEAK) has been approved to treat RRMS. Most studies have linked GAs’ potential to change T-cell differentiation to their immunomodulatory effects. It also acts on antigen-presenting cells which are involved in innate immunity [70].

Previous studies showed that GA therapy might be associated with improved fatigue. Ziemssen et al. used the MFIS scale and stated that treatment with GA is linked to a considerable reduction in absences from work and a reduction in fatigue symptoms over 12 months [40]. Ziemssen confirmed that fatigue and quality of life remains stable among 754 RRMS patients over 24 months of GA treatment [43]. In another study conducted by Metz et al. over 6 months using the FIS scale concluded that with GA treatment, the likelihood of decreased MS fatigue is roughly twice as high as with beta interferon [42].

Jongen et al. reported that in RRMS patients on GA treatment for two years, the improvements in fatigue seen after one year of treatment remained for the second year, and the levels of fatigue after two years were strongly linked with those at six and twelve months. In addition, mood and disability did not change from baseline [37]. Similarly, in 197 RRMS patients, GA treatment was linked to an improvement in HR-QoL in the first six months, which persisted at the 12th month. Among 10 individuals, 4 showed improvements in HR-QoL, which was linked to decreased fatigue [39]. In a study of 54 patients with moderate to severe fatigue switched from IFN-GA, participants experienced improvements in fatigue and overall better quality of life [41].

In a multicenter study using a cohort of 428 MS patients, 62.4% of 428 MS patients experienced fatigue. The degree of disability, fatigue, depression, and the length of illness all strongly correlated with quality of life [10]. Moreover, Neuhaus et al. reported that over the course of a year of GA treatment, 13 of 25 patients showed improvement on the three self-assessment measures (FSS, MS-FSS, and MFIS), while 5 patients showed worsening. The impacts experienced by the remaining seven patients varied across the three scales and showed inconsistent impacts across the three scales [38]. In a cross-sectional comparative study between various DMTs and in contrast to previous results, GA showed no improvement in fatigue among a total of 85 patients on either GA or interferon beta-1a. Neither GA nor interferon affected FSS. Surprisingly, there were no significant differences between these two classes of DMTs on their effect of MS-related fatigue [44].

### 6.2. Interferons

There are two different interferon drug classes: interferon-beta 1a and interferon-beta 1b. Avonex, Rebif, and Plegridy are examples of commercially available interferon-beta 1a types of drugs. The three most prevalent varieties of interferon-beta 1b are Betaseron, Betaferon, and Extavia. It is believed that both forms of interferons affect T cells, but because they target distinct cytokines, they can affect various cell cascades. Limited research studied the effect of interferon on fatigue.

In a study conducted by Melanson et al. on 50 patients with RRMS over 12 months using MFIS, fatigue improvement was observed in the subcutaneous interferon beta 1a group when compared to the intramuscular or interferon beta 1 b group [45].

An earlier prospective study utilizing interferon beta 1 A over 24 months revealed that 68.2%, 51.5%, and 57.4% of patients in the low-dose, high-dose, and dose escalation groups, respectively, had stable or improved fatigue according to the (FDS) scores at the 2-year follow up [46]. Approximately 70–100% of individuals receiving interferon (IFN) therapy report experiencing fatigue as a side effect. IFN-mediated fatigue (IMF) has a multifactorial etiology, with endocrine dysfunction, neuropsychiatric disorder, autoimmune, and cytokine dysregulation all having the potential to play a role. In 8–20% of individuals taking IFN-, thyroid impairment that is related to the formation of autoantibodies is observed. The hypothalamic–pituitary–adrenal axis is likewise suppressed by IFN. IFN therapy also causes cognitive slowness and depression, and depressed patients are more likely to experience fatigue [47].

## 7. Infusions

### 7.1. Natalizumab (NTZ)

Leukocyte migration across the blood–brain barrier (BBB) is decreased by natalizumab, a monoclonal antibody against Very-Late-Activation-Antigene 4. Because NTZ has a strong impact on CNS inflammation and marked reduced MRI lesions, we hypothesized that it may help lessen MS-related fatigue [71]. Putzki et al. conducted a prior prospective open-label uncontrolled trial over a 6-month period; MFIS and FSS were used. The researchers concluded that NTZ treatment improved fatigue [50]. Moreover, after conducting a one-year large sample prospective trial, Iaffaldano et al. found that NTZ resulted in a significant reduction in FSS scores [55]. Several prior trials used the FSMC, including those conducted by Svenningsson; in addition, Penner hypothesized that the use of NTZ reduces fatigue associated with MS based on the findings from a one-armed uncontrolled study [51,53].

In a cross-sectional case–control study, forty-nine patients who received NTZ treatment were compared to forty-three patients treated with IFN or GA. Both MFIS and FSS were used to measure fatigue. In comparison to the score of 4.9 ± 0.8 in those with IFN/GA, the mean FSS score was 3.7 ± 1.9 in those with NTZ (*p* = 0.012). No differences were found between IFN and GA. In comparison to 13.2% of IFN/GA patients, 51% of NTZ patients reported “no fatigue” (*p* < 0.001). In the NTZ group, the prevalence of severe fatigue was 34.7% compared to 51% in the IFN/GA group (*p* = 0.041) [54].

In another study where 48 patients with RRMS were included in the 3-year open-label, single group, multicenter clinical trial. Six months following the start of natalizumab therapy, the index of the MusiQoL, which measures global HRQoL, showed a substantial improvement compared to baseline, with a medium effect size (58.6 ± 16.2 vs. 69.8 ± 18.9, *p* < 0.001, Cohen’s d = 0.63). After three years, there was a negative correlation between the variation in fatigue score and the variation in global HRQoL (r = 0.44, *p* = 0.015). It also concluded that a greater baseline fatigue score was associated with an increase in overall HRQoL three years later (r = 0.34, *p* = 0.041). Natalizumab seemed to benefit mostly patients with more pronounced fatigue at baseline [52].

In contrast to the previous studies, Kunkel failed to find any significant improvement in fatigue among 51 RRMS NTZ-treated patients using FSMC scores over two years, suggesting that natalizumab has no effect on fatigue [57]. Interestingly, in a community-based sample of forty-eight RRMS patients (mean age = 38.3 years), MFIS was used to longitudinally assess MS-related fatigue. Over the course of the observation period of 12 months, worsening fatigue was found and the mean total MFIS score grew significantly from 32.6 ± 20.9 to 49.1 ± 20.0 (*p* < 0.001) compared to the previous year of treatment. The discrepancy between these results and results from other studies might be better explained by longer treatment duration in this study (30.4 ± 8.8 months), suggesting a continuous subtle inflammation in the CNS over time [56].

### 7.2. Alemtuzumab

Alemtuzumab, marketed under the brand name Lemtrada, has FDA approval for patients with active RRMS. It is a monoclonal anti-CD52 antibody that lowers the body’s T- and B-lymphocyte levels followed by reconstituting them [72].

In a study conducted by Wray et al., it was concluded that patient-reported fatigue, measured using MFIS-5, a significant decrease from a mean (SD) of 12.79 (4.98) at baseline to 10.96 (5.07) at Month 24 (*p* < 0.0001) was observed [48].

### 7.3. Ocrelizumab

MS, both relapsing and progressive, can be treated with ocrelizumab, which is a monoclonal antibody that works by binding to CD 20. A total of 98 RRMS and 32 progressive MS patients were administered a battery of patient-reported outcomes (PRO) over a 12-month period including SF-12 and Neuro-QoL. Significant improvement in fatigue was found with both SF-12 a Neuro-QoL [58].

### 7.4. Mitoxantrone

Mitoxantrone is an intravenous immunosuppressant that was indicated as useful for RRMS, PRMS and SPMS. It acts by the inhibition of both T and B cells as well as macrophage proliferation. Its use was limited by cardiotoxicity and myelosuppression [49]. Limited data were reported about its effect on fatigue. In a study conducted by Fox et al., the authors concluded that there is reported fatigue in six Mitoxantrone-treated patients. This may be explained by the fact that Mitoxantrone is used in more aggressive diseases [49].

## 8. Discussion

Since fatigue is one of the primary symptoms of MS, accurate assessment is necessary in daily clinical practice. However, the frequency and pattern of fatigue symptoms may differ from patient to patient and may also be affected by certain environmental and psychosocial factors. As a result, measuring fatigue is still a difficult endeavor. The complexity of this is increased by a variety of factors as there is no conclusive diagnostic laboratory test or biomarker. Unlike many other MS-related symptoms, some clinically obvious signs do not always indicate fatigue (e.g., paresis). Most importantly, it appears that fatigue has both physical and mental aspects. Despite the revolution in MS treatment, there is still limited data on how DMTs affect fatigue. Moreover, some studies using the same DMTs yield different outcomes [56,57]. This may be better explained by different disease population, EDSS and associated symptoms.

Notably, the most effective DMTs for reducing fatigue are natalizumab, GA and ponesimod. Out of eight studies exploring the effect of GA on fatigue, seven showed benefits, while one showed no effect. Natalizumab also showed promising results in reducing MS-related fatigue, with six studies showing positive effect, one showing neutral and one showing negative effect, but after a prior long duration of treatment with natalizumab, indicating subtle chronic inflammation, undefeated by natalizumab, as the main driver of MS fatigue. Additionally, in the only study examining the effect of ponesimod on the MS-related fatigue, ponesimod was associated with decreased fatigue when compared to teriflunomide in addition to reduced annualized relapse rate, magnetic resonance imaging activity, brain volume loss, and no evidence of increased safety concerns [31]. These results should be considered preliminary and interpreted with caution. Some of the studies were limited by their small sample, the lack of a control group and the short observation period. In addition, different scales were used, making the comparison of the results even more challenging. The use of DMTs should always take into consideration their side effects. Occasionally, fatigue was reported as a side effect of DMTs notably with interferons and fingolimod.

To better evaluate fatigue, the FDA has recently suggested using patient-reported outcome (PROs) as secondary and/or tertiary outcome measures in clinical trials. PROs are crucial for patients in real-world situations. The number of PRO measures that are currently in use has grown along with the appreciation of PROs’ worth. According to earlier research, there are over 400 published PROs altogether, and close to 100 of those are focused on MS.

While we cannot determine whether DMTs are friends or foes in improving MS fatigue, we hope this review article will serve as a stepping stone to increasing interest in the ways DMTs influence MS fatigue. Large, prospective, well-designed, head-to-head trials are still needed to further evaluate the effect of DMTs on fatigue and balance the associated safety profiles [73].

## Figures and Tables

**Table 1:** FDA-approved DMT and their methods of administration. DMT: Disease Modifying Therapies.

Oral DMTs	Infusion DMTs	Injectable DMTs
Dimethyl fumarate	Natalizumab	Glatiramer acetate
Sphingosine 1-P modulators (Fingolimode, Siponimod, Ponesimod, Ozanimod)	Alemtuzumab	Interferons
Teriflunomide	Ocrelizumab	Ofatumumab
Cladrabine	Mitoxantrone	

**Table 2:** Summary of various FDA-approved DMTs, mechanism of action and their effect on fatigue. SPMS: secondary progressive MS, PRMS: progressive relapsing MS, RRMS: relapsing remitting MS, CIS: clinically isolated syndrome, DMTs: disease modifying therapies. * 21 is the main study while 22 is a post hoc analysis. ** 40 showed mixed results.

DMT	Approved Indication	Mechanism of Action	Studies with Positive Impact on Fatigue	Studies with Negative Impact on Fatigue	Studies with No Impact on Fatigue
**Dimethyl fumarate**	CIS, RRMS	Activates the nuclear factor erythroid 2 related factor 2 (Nrf2) transcriptional pathway.	[13,14,15,16]		[17,18,19]
**Fingolimod**	CIS, RRMS	Sphingosine 1 phosphate receptor	[20,21,22] *	[23,24,25,26,27]	[28]
**Siponimod**	CIS, RRMS, active SPMS	Sphingosine 1 phosphate receptor		[29]	[30]
**Ponesimod**	CIS, RRMS	Sphingosine 1 phosphate receptor	OPTIMUM [31]		
**Teriflunomide**	CIS, RRMS	Mitochondrial dihydro-orotate dehydrogenase (DHODH) inhibitor	TENERE [32,33]	[34]	Teri-Fast [35]
**Cladribine**	CIS, active SPMS	Lymphocyte depletion	[36]		
**Glatiramer acetate (GA)**	CIS, RRMS	Anti-inflammatory	[37,38,39,40,41,42] **		[43,44]
**Interferons**	CIS, RRMS	Anti-inflammatory	[45,46]	[47]	
**Alemtuzumab**	Aggressive RRMS	Anti- CD20	[48]		
**Mitoxantrone**	RRMS, SPMS, PRMS	Type II Topoisomerase inhibitor		[49]	
**Natalizumab**	RRMS	Interfere with t cell transmigration	[50,51,52,53,54,55]	[56]	[57]
**Ocrelizumab**	RRMS, SPMS	Anti-CD20	[58]		

## Data Availability

Not applicable.
